# Diagnosis and treatment of urinary and sexual dysfunction in hereditary TTR amyloidosis

**DOI:** 10.1007/s10286-019-00627-7

**Published:** 2019-08-26

**Authors:** Imad Bentellis, Gérard Amarenco, Xavier Gamé, Dora Jericevic, Mehdi EL-Akri, Caroline Voiry, Lucas Freton, Juliette Hascoet, Quentin Alimi, Jacques Kerdraon, Benjamin M. Brucker, Benoit Peyronnet

**Affiliations:** 1 Department of Urology, University of Rennes, Service d’Urologie, 2 Rue Henri Le Guilloux, 35000 Rennes, France; 2 Department of NeuroUrology, Tenon Hospital, Paris, France; 3 Department of Urology, University of Toulouse, Toulouse, France; 4 Department of Urology and Obstetrics and Gynecology, New York University Langone Health, New York, USA; 5 Department of Physical Medicine and Rehabilitation, University of Rennes, Rennes, France

**Keywords:** TTR amyloidosis, Familial amyloidosis polyneuropathy type 1, Lower urinary tract dysfunction, Sexual dysfunction, Detrusor underactivity

## Abstract

**Purpose:**

We aimed to review the current knowledge on the epidemiology, diagnosis, and management of urinary and sexual dysfunction in patients with TTR amyloidosis (ATTR).

**Methods:**

We performed a review of the literature, screening for randomized controlled trials, prospective and retrospective series, position papers, and guidelines on urinary and sexual dysfunction in ATTR patients published in PubMed and Embase.

**Results:**

Lower urinary tract dysfunction is present in up to 83% of patients with ATTR. Voiding symptoms are the most common, reported in 34.8−87.5% of patients, while urinary tract infections are reported in up to 50%. Urinary incontinence is observed in 16.7−37.5% of the ATTR population, mostly due to decreased urethral resistance. Sexual dysfunction affects over 40% of ATTR patients, with erectile dysfunction and sexual arousal disorder being the most common symptoms in male and female patients, respectively. In addition to a thorough clinical examination, invasive pressure-flow urodynamic testing is a cornerstone in the assessment of ATTR lower urinary tract dysfunction. The most common finding is detrusor underactivity and intrinsic sphincter deficiency. Poor bladder compliance can also be observed in patients, due to amyloid deposits on the bladder wall. Urinary tract imaging may be of interest to rule out upper urinary tract deterioration. Given the paucity of data in the ATTR population, treatment should be tailored to the individual patient.

**Conclusion:**

Urinary and sexual dysfunction are highly prevalent in ATTR patients. Comprehensive assessment and multidisciplinary management are keys to avoiding upper urinary tract damage and improving patients’ quality of life.

## Introduction

First described in 1952 by Corino de Andrade, hereditary transthyretin amyloidosis (ATTR), formerly known as familial amyloid polyneuropathy type 1, is an autosomal dominant disease caused by a mutation in the transthyretin gene [1]. It is a rare disease, but its prevalence in endemic regions such as Portugal, Sweden, and Japan can exceed 1:1000 [2–5]. ATTR has an adult onset and can have lethal implications, with median survival of 10−15 years [3]. The transthyretin gene mutation causes a conformational transformation of transthyretin, a transport protein produced by the liver. The mutation results in the aggregation of transthyretin into amyloid fibrils that are deposited in various tissues and in peripheral and autonomic nerves, which results in organ dysfunction and progressive motor, sensory, and autonomic neuropathy. Among myriad clinical features, ATTR can result in lower urinary tract dysfunction (LUTD) and sexual dysfunction [3–7]. Owing to its relative rarity and heterogeneous clinical presentations, the diagnosis of ATTR is often delayed, compromising treatment, as tissue damage is largely irreversible, and current therapeutic options are mainly effective at early disease stages [8]. Beyond the impact of urinary and sexual symptoms on the quality of life of ATTR patients and possible improvement with appropriate management, awareness among the medical community of typical LUTD and sexual dysfunction associated with ATTR may improve early diagnosis [8]. The aim of the present study was to review the current evidence on urinary and sexual dysfunction in patients with TTR amyloidosis.

## Methods

**Fig. 1:**
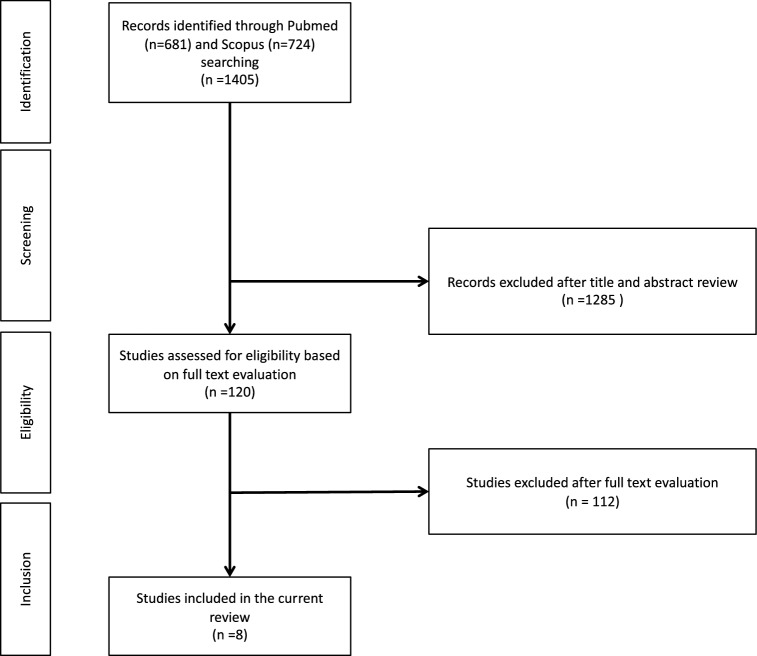
PRISMA flowchart of the studies selection process

A PubMed and Embase literature review was conducted in December 2018, screening for randomized controlled trials (RCTs), prospective and retrospective series, position papers, and guidelines on urinary and sexual dysfunction in ATTR patients. No time period or language restrictions were applied. There was only one article in Spanish, which was assessed by a native speaker. The literature was searched using the following terms alone or in combination: “TTR amyloidosis”, “ATTR”, “familial amyloidosis polyneuropathy type 1”, “TTR-FAP”, “V30 M”, “transthyretin”, “Corino de Andrade”, “dysautonomia”, “lower urinary tract dysfunction”, “lower urinary tract symptoms”, “bladder”, “detrusor”, “sexual dysfunction”. A total of 1405 records were screened for eligibility by title and abstract by two of the authors. After evaluation of 120 full-text manuscripts, only eight were included in the present analyses. A PRISMA [Preferred Reporting Items for Systematic Reviews and Meta-Analyses] flowchart detailing the study selection process is presented in Fig. 1.

## Results

### Epidemiology of urinary and sexual dysfunction in ATTR

The prevalence of lower urinary tract symptoms (LUTS) in ATTR patients is high, up to 83% [7] in this patient population. Recent data suggest that LUTD might occur at early disease stages [9]. However, LUTS severity and prevalence increase with increasing stage of disease [9]. Sexual dysfunction is also highly prevalent in ATTR patients, reportedly affecting up to 40% of male patients [5] and up to 42% of female patients [10]. Similar to LUTD, sexual dysfunction may be an early occurrence in disease progression [11]. Hence, LUTD and sexual dysfunction, if recognized by urologists, may favor early diagnosis of ATTR [10, 11]. The prevalence and severity of LUTD and sexual dysfunction seem to correlate strongly with overall autonomic dysfunction as assessed by the clinical evaluation scale–ATTR [10, 11]. However, data on specific associations between LUTD or sexual dysfunction and each autonomic dysfunction (e.g. peripheral neuropathy, cardiovascular dysfunction) are lacking.

### Pathophysiology

The pathogenesis of lower urinary tract and sexual dysfunction in ATTR is multifactorial and involves neurogenic, vasculogenic, and myogenic mechanisms [6–10]. The amyloid deposits in the endoneurium result in the loss of sensory somatic long fibers (peripheral thermalgesic sensation) and small myelinated Aδ fibers (autonomic regulation), both of which explain the findings of reduced bladder sensation. Autonomic neuropathy is a major contributor to ATTR-related LUTD, with impaired bladder afferent signaling and altered parasympathetic efferent output factoring in decreased bladder sensitivity and poor detrusor contractility. Sympathetic dysfunction from ATTR may also explain decreased proximal urethra resistance. Affection of somatic innervation may be a key determinant of intrinsic sphincter deficiency. The endothelial dysfunction resulting from extracellular deposition of amyloid fibrils favors chronic pelvic ischemia, a well-established factor of both bladder dysfunction and sexual dysfunction [11]. Amyloid deposits on the bladder wall could impair bladder compliance and disrupt suburothelial sensory function [3]. Although an increased prevalence of nocturia in the ATTR population has not been clearly shown, there may be specific pathophysiological mechanisms of nocturnal polyuria in these patients. Some studies have suggested that autonomic dysfunction could cause excessive inhibition of antidiuretic hormone (ADH) with recumbency, and excessive release of ADH when dysautonomic patients are up and about, resulting in both nocturnal polyuria and orthostatic hypotension, especially in the morning [12].

Psychologic factors may play an important role in sexual disorders in ATTR patients with dysautonomia, largely overlapping with other determinants of sexual dysfunction [13]. The pathophysiological mechanisms involved are similar to those of LUTD. Small myelinated afferent nerve loss contributes to decreased genital sensation (pressure, epicritic, vibrations) [14]. Vasculopathy reduces blood flow to the genital organs. Sexual hormone deficiency resulting from pelvic vasculopathy may also be a contributor to ATTR sexual dysfunction [3]. In women, denervation causes a decrease in sexual stimulation, clitoral blood flow, vaginal congestion/dilation, and lubrication. Psychological factors, coital incontinence, and pelvic organ prolapse may exacerbate symptoms [3].

### Clinical features

#### Lower urinary tract dysfunction

The terms used in this manuscript are in line with the International Continence Society report on the terminology for adult neurogenic lower urinary tract dysfunction (NLUTD) [15]. Data on LUTS in ATTR are relatively scarce and are summarized in Table 1 [6–10]. LUTD is seen mostly in patients with early-onset ATTR from endemic areas (Portugal, Japan, Sweden) and is much less prominent in late-onset sporadic cases from non-endemic areas [16]. Voiding symptoms appear to be the most common LUTS in these patients, with 34.8−87.5% reporting at least one voiding symptom (i.e. hesitancy and/or straining and/or intermittence) [6–10].

**Table 1:** Lower urinary tract symptoms in patients with TTR amyloidosis

	*n*	Age (years)	Gender	Duration from disease onset (years)	Voiding symptoms (%)	Incontinence (%)	Urinary tract infections (%)	Average daytime frequency	Average nocturnal frequency	Absent bladder sensation (%)	No urinary symptoms (%)
Andersson et al. [4]	8	52.25	100% Male	5.9	87.5%	37.5%	NR	NR	NR	37.5%	NR
Hita Villaplana et al. [5]	12	44	83.3% Male 16.7% Female	4.8	41.7%	16.7%	NR	NR	NR	NR	33.3%
Wada et al. [6]	21	38.7	76.2% Male 83.3% Female	NR	91%	38%	NR	NR	NR	NR	NR
Andrade et al. [7]	54	38	51.9% Male 48.1% Female	5.8	38.9%	24%	50%	6	0.83	3.7%	16.6%
Gomes et al. [9]	23	43.5% < 30 years 39.1% 30−40 years 17.4% > 40 years	100% Female	NR	34.8%	26.1%	21.7%	NR	NR	56.5%	69.6%

*NR* not reported

Underactive bladder is defined as a symptom complex suggestive of detrusor underactivity, typically characterized by prolonged urination time with or without a sensation of incomplete bladder emptying, usually with hesitancy, reduced sensation of filling, and a slow stream [17]. Underactive bladder may be a suitable term for designating NLUTD in most ATTR patients. Dysfunctional voiding with lack of urethral relaxation or even detrusor sphincter dyssynergia was observed in over half of patients in one series [7]. This finding would require further confirmation, as the pathogenesis of ATTR neuropathy would hardly explain such an NLUTD, and this feature was not observed in other series. Incomplete bladder emptying favors urinary tract infections, which are reported in up to 50% of ATTR patients [7]. Because of reduced bladder sensation, bacteriuria may remain asymptomatic in a significant proportion of patients with ATTR [10]. High post-void residuals also carry a risk of upper urinary tract damage, with some cases of ATTR patients with hydronephrosis having been reported [5]. Likely due to an over-representation of early disease stages in the series available in the current literature, the proportion of patients with chronic urinary retention was lower than that typically seen in daily practice in this patient population.

Urinary incontinence is another common occurrence in ATTR, reported in 16.7−37.5% of patients. Stress urinary incontinence (SUI) may be the predominant component of incontinence in most patients, with contribution of overflow incontinence in some of those with chronic urinary retention [6–10]. Other symptoms have been described, such as increased or decreased urinary frequency and urgency, but they appear to be much less common and probably less disease-specific than underactive bladder and stress urinary incontinence.

#### Sexual dysfunction

Like LUTD, sexual dysfunction is more common in early-onset ATTR than in late-onset cases, as part of the severe autonomic dysfunction observed in the early-onset population [16]. In men with ATTR, the most common sexual disorders are erectile dysfunction due to parasympathetic failure, reported in about 40% of patients, and retrograde ejaculation due to sympathetic failure (Table 2). Azoospermia has also been described, likely caused by amyloid deposits, with vasculopathy resulting in atrophy of the seminiferous tubules [18].

**Table 2:** Sexual symptoms in patients with TTR amyloidosis

	*n*	Mean age (years)	Mean duration from disease onset (years)	Sexual dysfunction (%)	Erectile dysfunction (%)	Retrograde ejaculation (%)	Sexual arousal disorder (%)	Dyspareunia (%)	Hypoactive sexual desire disorder (%)	Orgasmic dysfunction (%)	Lack of vaginal lubrication (%)
Female											
Gomes et al. [10]	23	< 30:10 30−40:9 > 40:4	NR	21.7%	NA	NA	NR	NR	NR	NR	33.3%
Oliveira-e-Silva et al. [19]	51	37.7	NR	42%	NA	NA	72.5%	39.2%	39.2%	62%	68%
1											
Hita Villaplana et al. [5]	10	44	4.8	NR	40%	10%	NR	NA	NA	NA	NA
Carr et al. [51]	10	62	2	NR	40.5%	NR	NR	NA	NA	NA	NA

*NR* not reported, *NA* not applicable

The prevalence of female sexual dysfunction ranges from 21.7 to 42% of ATTR patients [6, 9]. The most common sexual symptoms in women with ATTR are sexual arousal disorder (up to 72.5% of patients), hypoactive sexual desire disorder (up to 39.2%), lack of vaginal lubrication (up to 68%), orgasmic dysfunction (up to 62%), and dyspareunia (up to 39.2%). The prevalence and severity of female sexual dysfunction may be correlated with disease stage. Female sexual dysfunction is more common in ATTR patients than in healthy control populations [19].

### Assessment of urinary and sexual dysfunction in ATTR

#### Role of urinary and sexual symptoms in diagnosing ATTR

Early diagnosis of ATTR remains challenging owing to disease heterogeneity and lack of awareness among the medical community regarding this rare disease. Careful evaluation of urinary and sexual symptoms may help in diagnosing ATTR at an early stage, given that both have been shown to appear early after disease onset, especially in early-onset/endemic cases. The concept of red flags was recently introduced by Conceição et al. [8]. Transthyretin familial amyloid polyneuropathy is suspected when patients develop progressive peripheral sensory-motor neuropathy, associated with family history of neuropathy (94%) and/or autonomic dysfunction, or gastrointestinal disorder (40−48% in early onset). Cardiac hypertrophy, weight loss, carpal tunnel syndrome, renal failure, or ocular symptoms are less frequent (< 5% in early onset and < 10% in late onset) [20–23]. The diagnostic criteria for ATTR were settled by the European Network for TTR-FAP (ATTReuNET) [24]: at least two of the aforementioned symptoms and identification of amyloid deposits in tissue biopsies are required. Hence, urinary and sexual dysfunction, when occurring along with other clinical signs listed above, should prompt confirmatory diagnostic investigations such as tissue biopsy or genetic testing.

#### Lower urinary tract dysfunction evaluation

##### Clinical evaluation

A thorough medical history is of paramount importance as part of the LUTD evaluation in ATTR patients. Patients should be asked about their storage and voiding symptoms. Clinicians should look for other symptoms of the ATTR clinical spectrum such as sensory-motor neuropathy and autonomic dysfunction. A cough stress test should be performed to assess stress urinary incontinence. All sensations and reflexes in the urogenital area should be tested. A digital rectal examination should be performed to rule out benign prostatic hyperplasia or prostate cancer. Pelvic examination in women should look for pelvic organ prolapse or any pelvic mass. Questionnaires validated in neurogenic bladder populations such as the Qualiveen instrument may provide an accurate estimate of LUTS and assess their impact on patients’ quality of life [25]. The use of a voiding diary may objectify and gauge LUTS and is highly recommended in the initial evaluation of NLUTD.

##### Uroflowmetry and post-void residual

Uroflowmetry and post-void residual (PVR) measurement may be helpful in detecting voiding dysfunction, as a significant proportion of ATTR patients with incomplete bladder emptying may be paucisymptomatic owing to reduced bladder sensation [10]. Voiding patterns would be abnormal in over 50% of patients, with decreased maximum peak flow (MPF and APF) in up to 52.2% [9]. Most patients (69.6%) have plateaued or polyphasic uroflowmetry curves consistent with the diagnosis of detrusor underactivity. PVR can be significantly elevated in up to 21.7% of cases, increasing with disease stage [10].

#### Urinary tract imaging

##### Ultrasound

Because of its non-radiating, non-invasive nature and relatively low cost, ultrasound is generally the preferred imaging modality in ATTR patients with NLUTD. Beyond measuring PVR, ultrasound should look for upper urinary tract damage, as chronic urinary retention, a common occurrence in ATTR patients, may result in upper tract deterioration [26]. In this regard, ultrasound should rule out hydronephrosis and urolithiasis. In ATTR patients, ultrasonography has been described as a modality for evaluating spontaneous opening of the bladder neck at rest during storage, a pathognomonic finding of sympathetic failure [27]. In their series, Andrade et al. observed such spontaneous opening of the bladder neck in > 50% of patients, and more frequently in men. Thickening of the bladder wall (> 2 mm) was also observed in 42.6% of patients, also more frequently in men, and seemed to correlate with duration of disease. This bladder wall thickening may be the result of bladder outlet obstruction, but is most likely due to amyloid deposits on the bladder wall and could result in poor bladder compliance. In the series by Andrade et al., all patients with poor bladder compliance (*n* = 10) had a thickened bladder wall.

##### Computed tomography

In cases of upper tract abnormalities on ultrasound, contrast-enhanced computed tomography may be needed to provide a more detailed and accurate analysis of the urinary tract. Indeed, CT provides a more precise morphological analysis, particularly in the assessment of urolithiasis, by specifying the size, location, and density of the stones, which may influence therapeutic management. In the case of hydronephrosis, CT is more sensitive than ultrasound for identifying the cause of the obstruction, with better visualization of the ureter. CT is more sensitive for evaluating potential complications of upper tract UTIs overall. However, it is an irradiating exam, and therefore should not be the first-line option for routine urinary tract assessment. Indications for CT should be restricted to selected cases and should always be weighed against the burden of radiation exposure.

#### Urodynamics

Urodynamic testing is a key diagnostic tool in patients with neurogenic bladder for both initial assessment and follow-up. Ideally, video-urodynamic (i.e. urodynamic test with fluoroscopic imaging) rather than standard urodynamic testing should be performed in neurogenic patients [28]. The urodynamic findings reported in the ATTR literature are summarized in Table 3. Filling cystometry showed reduced bladder sensation in 38−56.5% of patients, with increased cystometric capacity in most series ranging from 432 to 670 mL on average [4–7, 9]. Poor bladder compliance was observed in 0−43% of patients, likely as a result of amyloid deposits on the bladder wall. Detrusor underactivity is defined by the International Continence Society as voiding contraction of reduced strength and/or duration, leading to prolonged or incomplete bladder emptying, and is the urodynamic correlate of underactive bladder [29]. The pressure-flow study found detrusor underactivity in 52.2−77.7% of patients and dyssynergia in 0−37.5%. Urethral pressure profilometry may demonstrate reduced maximum urethral closure pressure, which was observed in 18−71% of patients in the literature. Video-urodynamic testing can demonstrate open bladder neck and urethra at rest, usually considered a hallmark of autonomic neural deficit. This may also help to rule out detrusor sphincter dyssynergia.

**Table 3:** Urodynamic findings in patients with TTR amyloidosis

	No.	Reduced bladder sensation (%)	Cystometric capacity (mL)	Low bladder compliance (%)	Detrusor underactivity (%)	Dyssynergia (%)	Decreased maximum urethral closure pressure (%)	Mean peak urinary flow (mL/s)	Mean post-void residual (mL)
Andersson et al. [4]	8	NR	664	NR	75%	NR	NR	7.8	17
Hita Villaplana et al. [5]	12	NR	432	NR	NR	0%	NR	11.3	NR
Wada et al. [6]	21	38%	NR	43%	NR	19%	71%	NR	70.6
Andrade et al. [7]	54	NR	523.8	18.5%	77.7%	37.5%	18%	20.4	Men = 139 Women = 52
Gomes et al. [9]	23	56.5%	670	0%	52.2%	0%	NR	52.2%: < 12	17.4%: > 30

*NR* not reported

#### Urethrocystoscopy

Urethrocystoscopy may be helpful in providing a confirmatory diagnosis of TTR amyloidosis [30] through bladder biopsies and pathological examination. When ATTR diagnosis has already been confirmed by other tissue biopsies, urethrocystoscopy should not be part of the routine initial lower urinary tract assessment.

#### Renal function assessment

The estimated glomerular filtration rate should be assessed initially and over follow-up, as chronic urinary retention may result in chronic kidney disease, in addition to several other causes inherent in transthyretin amyloidosis. Serum creatinine should be used in patients with no/few motor impairments, while cystatin C should be favored in those with severe motor impairment/poor muscle mass [28, 31].

#### Sexual dysfunction evaluation

The basic workup should always include a medical and psychosexual history. The cardiovascular status should be determined to rule out high cardiovascular risk that is a common cause of sexual dysfunction, with prompt referral to a cardiologist when appropriate. The neurological status should also be established. The International Index of Erectile Function (IIEF) and the Female Sexual Function Index (FSFI) are the most widely accepted validated questionnaires for evaluating erectile dysfunction and female sexual dysfunction, respectively [32, 33]. In men, the physical examination should seek penile deformities and signs of hypogonadism. In women, the physical examination should look for pelvic organ prolapse, vulvovaginal atrophy, dermatological lesions, and tightness of pelvic floor muscle. In male patients, glucose lipid profile should be assessed if not done within the past 12 months, as well as serum total testosterone assessment, especially in cases of decreased libido. Laboratory evaluation is rarely helpful in guiding diagnosis or treatment of female sexual dysfunction.

### Treatment

As always in neuro-urology, the management of urinary and sexual dysfunction in patients with ATTR should ideally be multidisciplinary, involving urologists, neurologists, and physical medicine physicians, rehabilitation physicians, and physical and occupational therapists. The aims of this management should be to avoid upper urinary tract complications and to improve patients’ quality of life [28].

#### Lower urinary tract dysfunction

##### Underactive bladder

Several pro-contractile drugs have been investigated in the treatment of detrusor underactivity, but none has shown significant clinical benefits [34, 35]. At the early stage, when bladder sensation is reduced but with preserved detrusor contractility, timed voiding or Valsalva voiding may be useful. Patients should be carefully counseled though regarding the risk of pelvic organ prolapse in neurogenic patients with Valsalva voiding [36].

Clean intermittent catheterization (CIC) remains the standard of care in patients with detrusor underactivity and chronic urinary retention. However, the threshold PVR prompting initiation of CIC remains a matter of debate. Recent data and expert opinion suggest that conservative management without CIC could deliver satisfactory outcomes for PVR of up to 300−400 mL in asymptomatic patients without hydronephrosis on renal ultrasound [26, 37, 38]. In patients with symptomatic increased PVR and PVR > 300−400 mL, CIC should be initiated. Suprapubic tube may be an alternative to CIC in patients with motor impairment of the upper limbs, and should always be favored over indwelling urethral catheter owing to its reduced risk of urinary tract infection and urethral complications [39]. In selected patients with non-obstructive urinary retention, sacral neuromodulation might be offered as an alternative. There is no contraindication to the use of sacral nerve stimulation in the case of dysautonomia. CIC in a sitting position should be recommended owing to the common postural hypotension in ATTR patients. In very rare scenarios with the complete inability to perform CIC and long life expectancy, advanced reconstructive procedures such as continent catheterizable channel or ileal conduit might be offered in tertiary referral centers.

##### Stress urinary incontinence

Stress urinary incontinence in ATTR patients can result from the lack of sympathetic output to the bladder neck and inherent lack of urethral resistance, but also, in women, from common non-disease-specific mechanisms such as menopause or obstetrical trauma and inherent decrease in pelvic floor muscle tone. If the neurological affection of the pelvis is not too severe and pelvic floor muscle voluntary contractions are preserved, physical therapy with Kegel exercises is the most appropriate first-line treatment [40]. Duloxetine is a serotonin reuptake inhibitor that acts in Onuf’s nucleus, increasing the activity of pudendal motor neurons. This results in increased striated urethral sphincter tone and detrusor relaxation. Duloxetine is the only pharmacological treatment of stress urinary incontinence that has been proven effective in a placebo-controlled randomized trial in both men and women [41]. While recommended by several international guidelines, its use for stress urinary incontinence remains off-label in most countries [42]. There is no disease-specific contraindication to the use of duloxetine in patients with ATTR. When conservative measures fail, surgical treatment may be required. The surgical armamentarium for stress urinary incontinence in men includes bulking agents, slings, periurethral balloons (adjustable continence therapy), and artificial urinary sphincter devices. The anti-incontinence procedures in female patients include midurethral slings, bulking agents, fascial slings, periurethral balloons (adjustable continence therapy), and artificial urinary sphincter devices in some countries. No studies to date have reported the use of stress urinary incontinence surgery in patients with ATTR.

##### Low bladder compliance

Poor bladder compliance carries a high risk of upper urinary tract deterioration. Antimuscarinic agents can be offered as a first-line therapeutic option, with intradetrusor botulinum toxin injections being the second-line treatment in the case of failure. However, in ATTR patients with amyloid deposits in the bladder wall causing decreased bladder compliance, augmentation cystoplasty may, theoretically, be the only treatment with a reasonable chance of success. Cystectomy and ileal conduit could be discussed in patients with low bladder compliance and inability to self-catheterize. Of note, no studies so far have reported the outcomes of these treatments specifically in the ATTR population [43]. There is no absolute contraindication to the use of antimuscarinic agents in the case of dysautonomia. However, the severity of cardiac or digestive disorders must be the subject of multidisciplinary evaluation before treatment introduction. In all cases, close monitoring of tolerance and efficacy should be carried out. In the young ATTR patient population, the long-term use of these drugs and their possible association with cognitive decline should be weighed and discussed with patients before initiating treatment [44].

##### Nocturia

Nocturnal polyuria is the most common pathophysiological mechanism underlying nocturia [45]. Desmopressin, a synthetic analog of arginine vasopressin with antidiuretic activity, is usually regarded as the therapeutic option of choice to treat nocturia, several clinical trials having demonstrated that it results in reduced nocturnal urine output and nocturnal voids [45]. Desmopressin could be considered if evidence of nocturnal polyuria (i.e. nocturnal urine volume [including the first morning void]/24-h urine volume greater than 20% in young patients and greater than 33% in elderly patients [45, 46]) is noted on the voiding diary. Blood sodium levels should be monitored carefully, as severe hyponatremia may occur with desmopressin intake [45]).

##### Urinary tract infection

UTI prophylaxis should be individualized, as there is currently no prophylaxis protocol that is supported by high-level-of-evidence studies and that could be recommended without limitations [46]. Optimizing the lower urinary tract management should be the priority before considering any kind of antibiotic prophylaxis, as the later increases bacterial resistance and is not a relevant long-term option [46].

#### Sexual dysfunction

##### Male sexual dysfunction

Phosphodiesterase inhibitors are reportedly effective and safe in ATTR patients with erectile dysfunction [47]. There are insufficient data on the use of phosphodiesterase-5 inhibitors (PDE5i) in cases of autonomic disorders to formulate specific recommendations. However, PDE5i should be used cautiously in patients with ATTR, especially those with demonstrated orthostatic hypotension, as PDE5i have been shown to reduce blood pressure [48]. In cases where phosphodiesterase inhibitors fail, other options that could be offered for erectile dysfunction are vacuum erection devices, intraurethral alprostadil, intracavernous injections, and penile prosthesis.

For premature ejaculation, dapoxetine is the only pharmacological treatment approved by regulatory authorities. Other serotonin reuptake inhibitors, topical anesthetic agents, and tramadol can be used off-label in this indication. The combination of pharmacotherapy with psychological/behavioral therapies may optimize therapeutic outcomes [33].

##### Female sexual dysfunction

Cognitive behavioral therapy is the cornerstone of female sexual dysfunction management. Flibanserin, a novel non-hormonal therapy, recently became the first approved pharmacological treatment for women with hypoactive sexual desire disorder. Testosterone therapy is the only treatment supported by level 1 evidence for the management of sexual arousal disorder in women. In female patients with orgasmic disorder, directed masturbation is the preferred therapeutic approach. None of the treatments for female sexual dysfunction has been specifically assessed in patients with ATTR [32].

### Impact of ATTR treatments on sexual and lower urinary tract dysfunction

Currently, the two main therapeutic strategies for ATTR are liver transplantation and the transthyretin tetramer stabilizer tafamidis [24]. Data regarding the impact of the latter on urinary and sexual dysfunction are scarce. A recent case report suggests that tafamidis might result in mild improvement in some urodynamic outcomes such as bladder sensation and contractility [49].

One series has suggested that liver transplantation may improve LUTD and male sexual dysfunction in a significant proportion of patients [5].

#### Follow-up

As mentioned above, the risk of upper urinary tract deterioration is presumably lower in ATTR than in other neurological conditions such as spinal cord injury or spinal dysraphism. However, early diagnosis of NLUTD is paramount in any neurological management protocol to allow for early treatment when needed and to prevent irreversible urinary tract deterioration [46]. Therefore, early referral to a neuro-urologist for an initial clinical evaluation could be advocated even in asymptomatic patients. The neuro-urologist would then tailor the initial workup and follow-up to individual patients and symptoms. In most cases, annual follow-up visits are recommended in patients with neurourological disorders [46].

No routine urinalysis should be performed to screen for asymptomatic patients except in the case of planned invasive urological examination [46]. Asymptomatic bacteriuria is very common in patients with NLUTD, reaching 100% prevalence in those who self-catheterize [46]. However, current literature suggests that asymptomatic bacteriuria is not associated with the risk of urosepsis [50]. Hence, all scientific societies agree that asymptomatic bacteriuria should not be treated. Routine urinalysis would result in over-treatment of asymptomatic bacteriuria and increased bacterial resistance to antibiotics. Urinalysis should only be performed in symptomatic patients [46].

## Conclusion

Studies on urinary and sexual dysfunction in ATTR are scarce. Both disorders are highly prevalent in ATTR patients. Underactive bladder and stress urinary incontinence, underpinned urodynamically by detrusor underactivity and intrinsic sphincter deficiency, respectively, are the most common types of lower urinary tract dysfunction. Erectile dysfunction and sexual arousal disorder are the most common sexual dysfunction in male and female patients, respectively. Comprehensive assessment and multidisciplinary management are keys to improving the quality of life of ATTR patients suffering from urinary and/or sexual dysfunction, and preventing upper urinary tract damage.
